# Left Ventricle Detection from Cardiac Magnetic Resonance Relaxometry Images Using Visual Transformer

**DOI:** 10.3390/s23063321

**Published:** 2023-03-21

**Authors:** Lisa Anita De Santi, Antonella Meloni, Maria Filomena Santarelli, Laura Pistoia, Anna Spasiano, Tommaso Casini, Maria Caterina Putti, Liana Cuccia, Filippo Cademartiri, Vincenzo Positano

**Affiliations:** 1 Department of Information Engineering, University of Pisa, 56122 Pisa, Italy; lisa.desanti@phd.unipi.it; 2 U.O.C. Bioingegneria, Fondazione G. Monasterio CNR-Regione Toscana, 56124 Pisa, Italy; antonella.meloni@ftgm.it; 3 Department of Radiology, Fondazione G. Monasterio CNR-Regione Toscana, 56124 Pisa, Italy; laura.pistoia@ftgm.it (L.P.);; 4 CNR Institute of Clinical Physiology, 56124 Pisa, Italy; santarel@ifc.cnr.it; 5 Unità Operativa Semplice Dipartimentale Malattie Rare del Globulo Rosso, Azienda Ospedaliera di Rilievo Nazionale “A. Cardarelli”, 80131 Napoli, Italy; 6 Centro Talassemie ed Emoglobinopatie, Ospedale “Meyer”, 50139 Firenze, Italy; 7 Clinica di Emato-Oncologia Pediatrica, Dipartimento di Salute della Donna e del Bambino, Azienda Ospedale Università, 35128 Padova, Italy; 8 Unità Operativa Complessa Ematologia con Talassemia, ARNAS Civico “Benfratelli-Di Cristina”, 90127 Palermo, Italy

**Keywords:** cardiac magnetic resonance, left ventricle, deep learning, object detection, visual transformer

## Abstract

Left Ventricle (LV) detection from Cardiac Magnetic Resonance (CMR) imaging is a fundamental step, preliminary to myocardium segmentation and characterization. This paper focuses on the application of a Visual Transformer (ViT), a novel neural network architecture, to automatically detect LV from CMR relaxometry sequences. We implemented an object detector based on the ViT model to identify LV from CMR multi-echo T2* sequences. We evaluated performances differentiated by slice location according to the American Heart Association model using 5-fold cross-validation and on an independent dataset of CMR T2*, T2, and T1 acquisitions. To the best of our knowledge, this is the first attempt to localize LV from relaxometry sequences and the first application of ViT for LV detection. We collected an Intersection over Union (IoU) index of 0.68 and a Correct Identification Rate (CIR) of blood pool centroid of 0.99, comparable with other state-of-the-art methods. IoU and CIR values were significantly lower in apical slices. No significant differences in performances were assessed on independent T2* dataset (IoU = 0.68, *p* = 0.405; CIR = 0.94, *p* = 0.066). Performances were significantly worse on the T2 and T1 independent datasets (T2: IoU = 0.62, CIR = 0.95; T1: IoU = 0.67, CIR = 0.98), but still encouraging considering the different types of acquisition. This study confirms the feasibility of the application of ViT architectures in LV detection and defines a benchmark for relaxometry imaging.

## 1. Introduction

**Figure 1:**
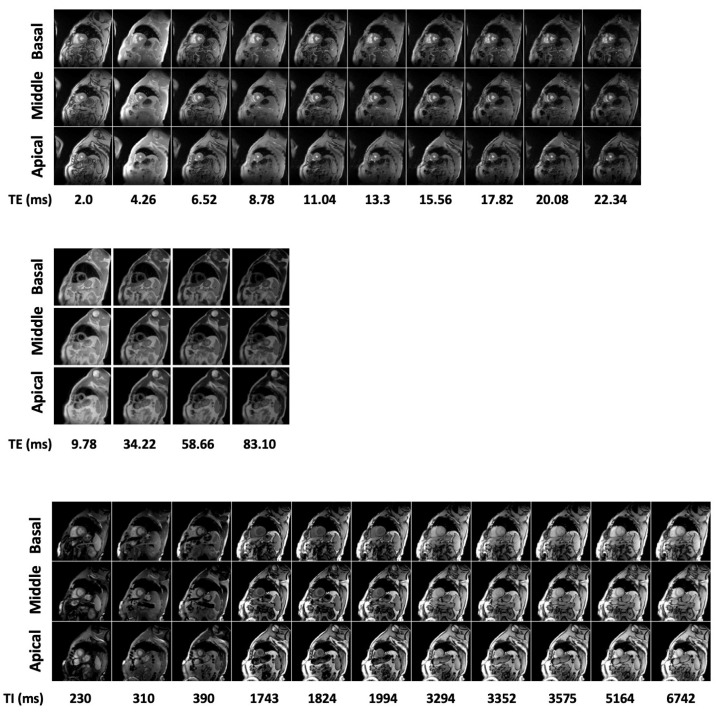
Examples of T2* (upper panel), T2 (middle panel) and T1 (lower panel) CMR image sequences.

Cardiac Magnetic Resonance (CMR) represents the gold standard non-invasive imaging tool for quantifying cardiac anatomy and function, as well as performing myocardial tissue characterization [1]. The Left Ventricle (LV) contraction is the source for the distribution of oxygenated blood to the entire body; hence, CMR image acquisition and analysis are focused on LV. In particular, the analysis of cardiac Short Axis Views (SAV) covering the Left Ventricle (LV) is mandatory in CMR to assess global and segmental cardiac function, myocardial perfusion, late gadolinium enhancement, and to characterize the myocardial tissue by T1, T2, and T2* relaxometry [2]. SAV planes are perpendicular to the long axis of LV that intercepts the apex and the center of the mitral valve, so that SAV planes preserve the integrity of the cardiac chambers, allowing us to perform standardized segmentation of the myocardium according to the segmental model defined by the American Heart Association (AHA) guidelines [3]. Image analysis of SAV series can be performed manually or by semi-automatic or fully-automatic algorithms. The use of automatic algorithms is strongly desirable to reduce processing time and avoid intra- and inter-subject variability [4]. SAV series are acquired over a large field of view, including the whole patient’s body, to avoid aliasing artifacts (Figure 1). Consequently, a typical pre-processing step in SAV analysis is a cropping operation centered on the object of interest (i.e., LV). Accurate identification and location of the LV in the SAV represent a pre-requisite for automatic evaluation of cardiac function indices, as this step provides good initialization for segmentation methods by extracting an ROI around the myocardium and cropping the initial volume ignoring all the other organs [5,6,7,8]. LV region extraction was also demonstrated to be useful in ischemic scar detection [9] and myocardial perfusion quantization [10]. In MR relaxometry, limiting the MR signal fitting to the LV region would allow a significant reduction in the required processing time [11,12].

In Computer Vision (CV) applications, the identification of LV represents an Object Detection (OD) problem, where the object detected will be marked using a Region Of Interest (ROI) such as a bounding box. The identification of the LV blood pool cavity from SAV images is a nontrivial task for different reasons [4]: (i) misalignment of SAV slices; (ii) inter-patient variability of LV cavity location; (iii) inter and within slices variability of the blood signal intensity (due to coil sensitivity variations and cardiac flow dynamics, respectively); (iv) presence of similarly shaped and isointense structures.

Object detection methods can be divided into two main classes: handcrafted-based and Deep Learning (DL)-based approaches [13].

Handcrafted approaches refer to the class of conventional algorithms based on mathematical models designed by field experts using domain knowledge [13,14]. Some examples in LV detection on fast-cine sequences include the usage of geometrical-based techniques such as a combination of scout-view geometry, blood-to-myocardial tissue contrast, and geometrical continuity constraint [4,5]; motion information obtained using Fourier transform [6,15,16]; or the usage of harmonic images to produce edge maps followed by anisotropic weighted circular Hough transform [17]. All the works mentioned have been applied to fast-cine MR images using internal or publicly available datasets.

At present, Artificial Intelligence (AI) is increasingly applied in the field of medical imaging analysis to help physicians in solving diagnostic problems and improve efficiency. DL models are a subset of AI-based algorithms that can be trained to learn discriminant features to perform a certain task automatically from data. There are several studies that deal with LV identification based on deep learning algorithms from CMR images. Abdeltawab et al. proposed a novel DL approach for the automated segmentation and quantification of the LV from single frames of fast-cine CMR images [18]. The authors developed and validated their model using both a locally acquired and a public dataset collected from the Automated Cardiac Diagnosis Challenge (ACDC-2017). Their framework started with a localization of the LV blood pool center point using a Fully Convolutional Neural Network (FCN) architecture to extract an ROI that contains the LV from all heart sections. Niu et al. developed a model to detect myocardium from the CMR image series from Cardiac Atlas Project (CAP) dataset. The model consists of a region proposal followed by a deep-Stacked Sparse Auto-Encoder (SSAE), and finally, a candidate regions classification and regression to refine the location of myocardium [19].

Region-based Convolutional Neural Networks (R-CNN) are a particular class of DL models commonly employed in OD tasks. They can be distinguished into (i) two-stage detection framework, e.g., R-CNN, Fast R-CNN, Faster R-CNN, Feature Pyramid Network (FPN); (ii) and one-stage detection framework, e.g., You Only Look Once (YOLO), Single Shot Detector (SSD), CenterNet and EfficientDet [20]. Wang et al. proposed a CNN model for LV detection in cardiac MRI slices from the CAP dataset by combining discriminative dictionary learning and sequence tracking. They compared their proposed model with different R-CNN models (Faster R-CNN, SSD, and Yolov3) to investigate the model’s performance [21]. Shaaf et al. [8] also used an R-CNN, in particular Faster R-CNN, to detect LV from SAV images using the Sunnybrook Cardiac Dataset (SCD). The network takes as input a single slice of a selected frame of a Steady-State Free Precession (SSFP) sequence.

Despite the incredible success achieved by CNNs, interesting performances in several CV tasks have been performed for another architecture: the so-called Vision Transformers (ViTs) [22,23,24]. Transformers were originally introduced in the field of Natural Language Processing (NLP), where they achieved state-of-the-art performances. Inspired by the incredible success achieved in NLP, researchers started to apply transformers directly to images, generating the ViT models [22]. ViTs can overcome some of the major shortcomings of CNN architectures, such as the inability to model long-distance spatial relationships and the overreliance of CNNs on textures with a weakness in modeling shapes. Due to their local receptive field, CNNs have a limited ability to capture long-range spatial dependencies in an input image while ViTs use an alternative architectural design able to capture the global context of an input image and can easily model long-distance visual relationships. In addition, the ability to function based on shapes rather than on textures is also highly attractive and promises better generalizability and robustness [25]. ViTs’ architecture includes a new attention-driven building block, which is a neural network layer that aggregates information from the entire input sequence, exploiting the so-called *“self-attention (SA) mechanism”* [23]. We can identify several ViT architectures for OD, some based on “pure” transformer models (only ViT layers), and others on a hybrid framework (both convolutional and transformer layers), e.g., DEtection TRansformer (DETR), COnvolutional and TRansformer layers (COTR) [23].

It should be emphasized that at this time it is unclear if the level of generalization and performance improvement that is seen in NLP applications with large networks and large datasets will also emerge in medical imaging applications. Many questions regarding the optimal architecture are unresolved, and we are truly just at the beginning of this exploration [25]. Finally, to the best of our knowledge, only a few attempts have been made in applying transformer models in medical image detection [23] and no one applied them to CMR relaxometry sequences.

In this work, we implemented an object detector architecture based on ViT model [22] to automatically detect LV from CMR multi-echo T2* sequences collected from the Italian Myocardial Iron Overload in Thalassemia (MIOT) Network [26]. We also tested the detector’s performances using as input an independent test set of T2*, T2, and T1 sequences to check its generalizability power also in different relaxometry acquisitions.

## 2. Materials and Methods

### 2.1. Study Population

Baseline images from 530 patients were retrospectively studied. All patients were consecutively enrolled from 2009 to 2020 in the core center of the MIOT/eMIOT (Myocardial Iron Overload in Thalassemia) network. This network is constituted by thalassemia and MRI centers where MRI exams are performed using homogeneous, standardized, and validated procedures and where patients’ clinical-instrumental data are collected in a centralized, web-based database [26,27]. The inclusion criterion was the availability of a CMR T2* multiecho sequence that is mandatory in the study protocol and was available in all patients. Twenty patients enrolled in an ancillary study with T2*, T1, and T2 mapping sequences available were also added to the study to serve as the external test set. The study complied with the Declaration of Helsinki. All subjects gave written informed consent to the protocol. The project was approved by the institutional ethics committee.

### 2.2. MR Imaging

MR exams were performed on 1.5T scanners (GE Signa/Excite HD, Milwaukee, WI, USA) using a cardiac phased-array receiver surface coil for signal reception. Three parallel short-axis views (basal, middle, and apical) of the LV were obtained by a T2* gradient–echo multiecho sequence (matrix = 256 × 256, FOV = 35 × 35 cm, thickness = 8.0 mm) with electrocardiogram triggering. Each single SAV was acquired at ten echo times (TEs 2.0–22 ms with an echo spacing of 2.26 ms) in a single end-expiratory breath-hold [28]. For T2 mapping, three parallel short-axis slices (basal, mid-ventricular, and apical) of the LV were acquired in end diastole by a Multi-Echo Fast-Spin-Echo (MEFSE) sequence (matrix = 256 × 256, the field of view—FOV = 35 × 35 cm, thickness = 8.0 mm) with 4 echo times (TEs): 9.78, 34.22, 58.66, and 83.10 ms. A black blood-prepulse (double-inversion-recovery) was applied [29]. For T1 mapping, three parallel short-axis slices (basal, middle, and apical) of the LV were acquired in end-diastole by a Modified Look-Locker Inversion Recovery (MOLLI) sequence (matrix = 256 × 256, FOV = 35 × 35 cm, thickness = 8.0 mm) with a 3 (3 s) 3 (3 s) 5 scheme In-line motion correction was applied to MOLLI images [30]. Figure 1 reports typical T2*, T2, and T1 image sequences.

### 2.3. Ground Truth

T2* image analysis was performed by trained MRI operators using a custom-written, previously validated software (HIPPO MIOT^®^) [28]. Endocardial and epicardial contours were manually traced on the three slices at the TE value providing a better contrast between myocardium and surrounding tissues and propagated among the other frames. The myocardium defined in the previous step was automatically segmented into equiangular segments. Six segments were used in the basal and middle slices, and four were used in the apical slice. For each segment, the mean value of the signal intensity along all TE values was calculated and fitted with a single exponential decay model, obtaining the corresponding T2* segmental value. The global heart T2* value was obtained by averaging all 16 segmental T2* values, according to the standard AHA model [3]. The assessed global T2* values ranged from 2 to 57 ms (mean 37.8 ± 8.97 ms), covering the entire spectrum of clinically relevant T2* values. Ground Truth (GT) bounding boxes were generated by creating the least rectangular region of interest that contains the entire epicardial mask. Each bounding box was defined by the (x,y) location of the top left and bottom right corners, normalized for the image size. As the correct detection of the Blood Pool (BP) region is important in several applications, the ground truth BP was defined as the center of the bounding box containing the endocardial contour. A modified version of the HIPPO MIOT^®^ software was used to define endocardium and epicardium in T2 and T1 sequences following a similar approach.

### 2.4. ViT Architecture

We selected the ViT architecture developed by Dosovitskiy et al. [22], substituting the final classification layer with a regression layer to predict bounding box coordinates. This section focuses on the description of the basic ViT components [23].

A ViT splits the input image into patches, then flattens and projects them into a positionally-embedded feature space. An encoder subsequently processes the embedded input to produce the final output. The transformer encoder implements the SA mechanism to determine the relative importance of a single embedded patch with respect to all others in the input. ViT uses a channel-wise concatenation of multiple SA blocks to model complex dependencies between the different elements of the input [23].

**Figure 2:**
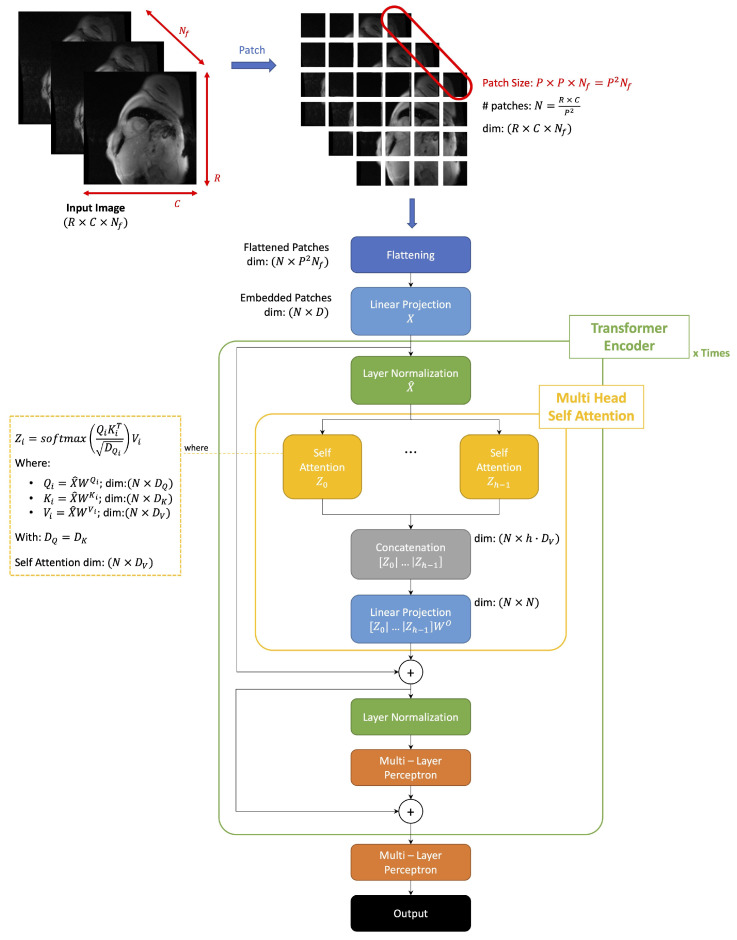
Visual transformer general architecture. Our model receives as input multi-echo CMR sequences and predicts a bounding box to detect the left ventricle.

In Figure 2, we reported an overall scheme of the operation performed by the model implemented.

Given a certain 2D image sequence:

(1) $$x\in R^{R\times C\times N_{f}}$$

ViT splits it into patches of fixed sizes and vectorizes them using a flattening operation. We obtain a sequence of flattened 2D patches:

(2) $$x_{P}\in R^{N\times (P^{2}N_{f})}$$

where

- $R\times C\times N_{f}=Rows\times Columns\times N_{frames}$ of the original input image
- P×P: size of a patch
- $N=\frac{RC}{P^{2}}$: resulting number of patches
- $P^{2}N_{f}$: dimension of flattened patch

ViT next applies a linear projection to the flattened patches to create a low-dimensional linear embedding. The weights of this linear layer are learned during the training phase. We obtain a linear embedding of the sequences of flattened patches, which, are furthermore positionally encoded:

(3) $$X\in R^{N\times D}$$

where *D* is the dimension of the embedded space.

The embedded patches are sent into the transformer encoder block, which implements the Multi-Head Self Attention (MHSA) mechanism composed of multiple SA blocks. A single transformer’s architecture can be constituted by a sequence of multiple transformer blocks connected in a cascade.

The SA mechanism captures the interaction between all the N embeddings to determine the importance of each embedded patch with respect to all the others.

The input sequence of patches *X* is projected into three different spaces:

- Queries: $Q=XW^{Q}$
  - $W^{Q}\in R^{D\times D_{q}}$
- Keys: $K=XW^{K}$
  - $W^{K}\in R^{D\times D_{k}}$
- Values: $V=XW^{V}$
  - $W^{V}\in R^{D\times D_{v}}$

All the weights matrices $W^{Q},W^{K},W^{V}$ are matrices of learnable weights and $D_{q}=D_{k}$.

Then, we use this projection to generate the corresponding attention matrix and compute the output of a SA layer.

Attention Matrix, $A\in R^{N\times N}:$ 

(4) $$A=softmax\left(\frac{QK^{T}}{\sqrt{D_{q}}}\right)$$

Output of the SA layer, $Z\in R^{N\times D_{v}}$ 

(5) Z=AV

An MHSA block is composed of a channel-wise concatenation of multiple SA blocks $\left[Z_{0},Z_{1},\ldots ,Z_{h-1}\right]$. Each SA block (head) has its learnable weight matrices $\left\{W^{Q_{i}},W^{K_{i}},W^{V_{i}}\right\}$ with i=0,…,(h−1) and where *h* denotes the number of heads in the MHSA block.

The MHSA block returns as the final output a linear projection of the multiple heads:

(6) $$MHSA\left(Q,K,V\right)=\left[Z_{0},Z_{1},\ldots ,Z_{h-1}\right]W^{O}$$

where $W^{O}\in R^{h\cdot D_{v}\times N}$ computes linear transformation of heads and a single head $Z_{i}$ can be written as:

(7) $$Z_{i}=softmax\left(\frac{XW^{Q_{i}}{\left(XW^{K_{i}}\right)}^{T}}{\sqrt{D_{q}/h}}\right)XW^{V_{i}}$$

Each transformer encoder block implements first a layer normalization followed by an MHSA operation.

Then, the output of the MHSA is added to the input of the normalization layer via a skip connection. The output of the skip-connection is further normalized and sent to a Multi-Layer Perceptron (MLP) block (multiple layers of fully connected units). Finally, the output of the MLP is added to the input of the last normalization layer via a skip connection.

The output of the transformer encoder goes to an MLP which returns as output the four dimensions representing the bounding box coordinates of an object, in our case the (x,y) coordinates of the top left and bottom right angles of the bounding box.

### 2.5. Model Training and Validation

We selected the first, fifth, and ninth echo times from the original sequence to train the network. Each scan given as input to the network is composed of 256×256 slices at the 3 different selected echo times; hence, the input image sequence dimensions are R=256, C=256, $N_{f}=3$. No further pre-processing operations were applied in input sequences.

We selected the ViT architecture from the Keras implementation available on GitHub trained on the Caltech 101 dataset [31]. We selected this architecture as it results in a reduced number of parameters to be optimized if compared to the original ViT-Base version [22]. This may help in preventing overfitting considering the limited dimension of our dataset. The model is characterized by the following hyperparameters:

- Patch size p=32 (resulting #patches N=64);
- Dimension of embedded space D=64;
- #heads in each MHSA layer h=4 with dropout =0.1;
- An MLP in each transformer encoder with 2 layers of, respectively, 128 and 64 units and dropout =0.1;
- A final MLP with 5 layers of, respectively, 2048, 1024, 512, 64, and 32 units and dropout =0.3.

To avoid data leakage problems we split the dataset into training, validation, and test sets so that basal, middle, and apical slices of the same subjects go into the same set [32].

The entire dataset employed is composed of 1590 images (3 slices of 530 different subjects). We used 10% and 20% as respective validation and test split, resulting in 1143 images in the training set (3 slices of 381 different subjects), 129 in the validation set (3 slices of 43 different subjects), 318 in the test set (3 slices of 106 different subjects). We applied on-the-fly data augmentation on the training set using random shift along both the x and y axes (range [−20÷20] pixels). We followed a stratified k-fold cross-validation (k = 5) strategy for the training + validation/test split and we reported the average performances on the test set over the 5 folds.

We trained from scratch our neural network by using an Adam optimizer to minimize the 1−DICE index between the GT and the predicted bounding box as the loss function. We used a mini-batch strategy with batch size =32. We set an initial learning rate of $10^{-4}$ and we decreased it during the training with a decay rate =0.96 and a decay steps =100,000. We set a maximum number of epochs =300. All the other optimizer’s parameters were left at the default value.

We evaluated the model’s performances over the 5 folds on the test set, computing the following metrics:

- Intersection over Union (IoU) or Jaccard Index:
  (8) $$IoU=\frac{|BB_{GT}\cap BB_{pred}|}{|BB_{GT}\cup BB_{pred}|}$$
- DICE Index:
  (9) $$DICE=2\frac{|BB_{GT}\cap BB_{pred}|}{|BB_{GT}|+|BB_{pred}|}$$
  where $BB_{GT}$ and $BB_{pred}$ represent, respectively, the ground truth and predicted bounding boxes.
- Centre Point Absolute Error, $\epsilon_{CP,A}$: Euclidean distance between the center point of the ground truth and predicted bounding boxes;
- Centre Point Epicardial Fractional Error, $\epsilon_{CP,epi}$: $\epsilon_{CP,A}$ normalized by the radius of a circle having the same area as the epicardial mask;
- Centre Point Endocardial Fractional Error, $\epsilon_{CP,endo}$: $\epsilon_{CP,A}$ normalized by the radius of a circle having the same area as the endocardial mask;
- Correct Identification Rate, CIR: Rate that the Centre Point of the predicted bounding box falls within the endocardial mask on the entire test set.

We selected the best model obtained over the five folds and we evaluated the performances on the three different independent test sets.

### 2.6. Statistical Analysis

The normality of the distribution of the parameters was assessed by using the Kolmogorov–Smirnov test or the Shapiro–Wilk test for a sample size ≤ 50.

One-way repeated measures ANOVA or the Friedman test were used to evaluate if there was a significant difference among parameters in different slices and the Bonferroni adjustment was used in all pairwise comparisons.

For continuous values with normal distribution, comparisons between groups were made by independent-sample *t*-test (for 2 groups) or one-way ANOVA (for more than 2 groups). Wilcoxon’s signed rank test or Kruskal–Wallis test were applied for continuous values with non-normal distribution. The Bonferroni post hoc test was used for multiple comparisons between pairs of groups.

We also evaluated if performances in the independent T2*, T2, and T1 series significantly differ compared to the ones reported on the test set considering the best fold.

In all tests, a 2-tailed probability value of p=0.05 was considered statistically significant.

### 2.7. Hardware and Software Specification

The proposed deep learning model was implemented using Python utilities (version 3.9), with Keras framework on the Tensorflow backend (version 2.6.0). The training was performed on an Intel Core i7 5.1 MHz PC, 32 Gb RAM, equipped with an NVIDIA RTX3090 GPU with 24 Gb of embedded RAM. Developed code is available at https://github.com/desantilisa/ViT_LeftVentricle_Object_Detection (accessed on 26 January 2023).

Statistical analysis was performed using the SPSS version 27.0 statistical package.

## 3. Results

**Figure 3:**
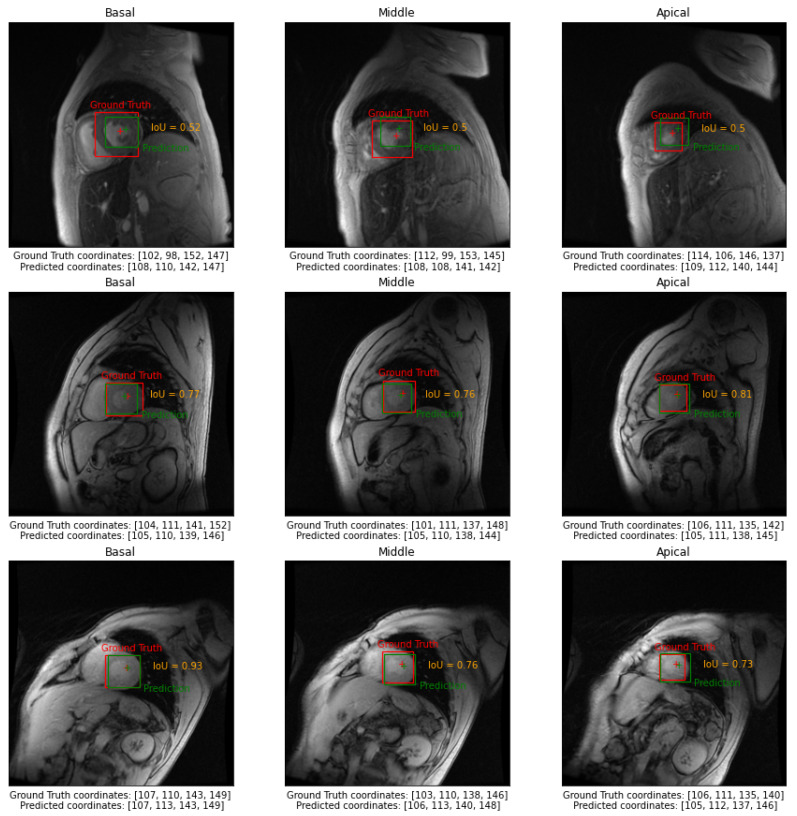
Examples of the model’s output in three different subjects belonging to the test set in basal, middle, and apical slices.

**Figure 4:**
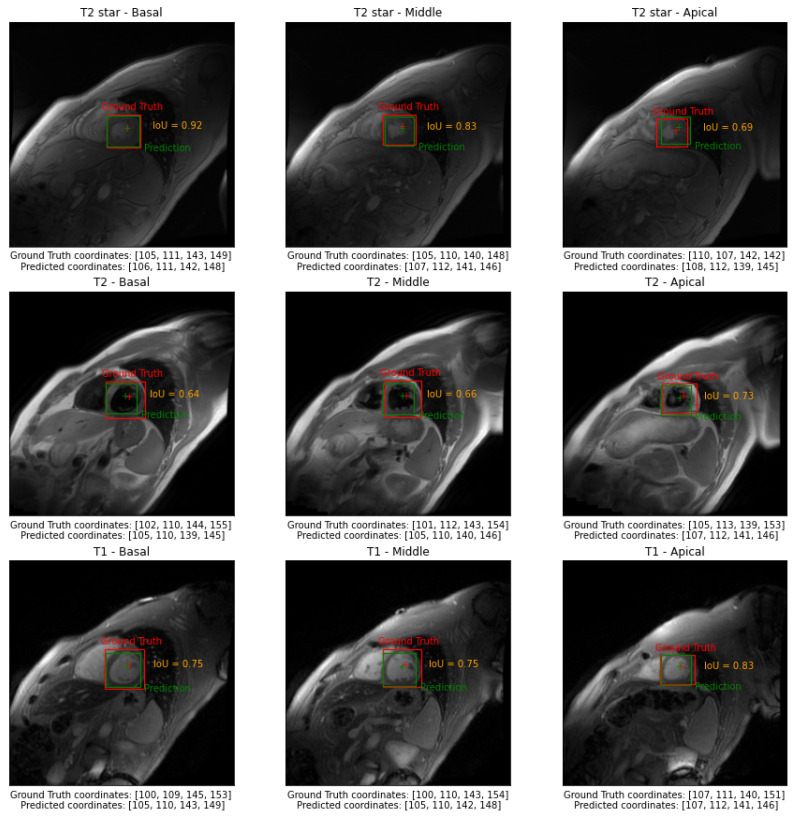
Examples of the model’s output of a selected subject for all three independent test sets in basal, middle, and apical slices.

In Figure 3, we reported examples of the model’s output of three different subjects belonging to the test set in basal, middle, and apical slices. In Figure 4, we reported examples of the model’s output of a selected subject for all three independent test sets in basal, middle, and apical slices.

In Table 1, we reported the averaged performances evaluated in the test set over the five folds differentiated by short-axis views of the AHA model (basal, middle, and apical) and the global ones. Averaged training times was 1 h ± 5 min; averaged single prediction time once the network was trained was 20 ± 4 ms.

In Table 2, we reported the averaged performances over the test set using the model that achieves the best performances (fold #1). Averaged performances on the independent T2*, T2, and T1 test sets were also reported. Statistical difference in performances between slices was observed in the test set between apical vs. basal and apical vs. middle IoU, DICE, $\epsilon_{CP,epi}$ and $\epsilon_{CP,endo}$ with a reported p≤0.0001 in all pairwise comparisons. No relevant difference in performances was observed according to slice type in the T2* independent test sets (IoU, p=0.405; DICE, p=0.405; $\epsilon_{CP,A}$, p=0.495, $\epsilon_{CP,epi}$, p=0.066; $\epsilon_{CP,endo}$, p=0.066), T2 independent test sets (IoU, p=0.827; DICE, p=0.827; $\epsilon_{CP,A}$, p=0.867, $\epsilon_{CP,epi}$, p=0.097; $\epsilon_{CP,endo}$, p=0.066), and T1 independent test sets (IoU, p=0.717; DICE, p=0.717; $\epsilon_{CP,A}$, p=0.717, $\epsilon_{CP,epi}$, p=0.565; $\epsilon_{CP,endo}$, p=0.172).

The statistical analysis highlighted no significant difference between performances in the test set and the T2* independent test set. We reported a significant difference in IoU basal and middle (p<0.0001), DICE basal and middle (p<0.0001), and $\epsilon_{CP,A}$ in all three slice types in the test set and T2 independent test set (basal: p=0.024, middle: p<0.0001, apical: p=0.036). Differences in IoU basal (p=0.024) and DICE basal (p=0.024) were also observed in the test set and T1 independent test set.

## 4. Discussion

In our study, we applied a ViT architecture to automatically localize the LV blood pool from CMR multi-echo T2* relaxometry sequences collected from the Italian MIOT. We also tested the detector’s performances in an independent test set of different relaxometry sequences to check its generalization capability to different types of acquisition.

Transformer networks are novel architectures that obtained state-of-the-art performances in NLP tasks. Due to their appealing properties, these models are gaining ground even in the medical imaging community; however, at present, there are only a limited number of applications that perform OD tasks in this field. To the best of our knowledge, this is the first attempt to train a ViT network to localize LV from CMR relaxometry sequences [23,25].

LV detection is an important step to facilitate subsequent operations to perform quantitative measures of myocardial structure, anatomy, and function. We found several works in the literature that deal with the detection of LV from fast-cine CMR using different strategies exploiting both handcrafted and deep learning-based approaches. A direct comparison of our results with the ones reported in the literature is difficult, as most of the studies are focused on LV segmentation, so the efficiency of the preliminary LV location task was not quantitatively assessed. Other recent studies focused on heart structure localization do not include comparison with ground truth data as in [33]. We selected the studies that, to our knowledge, assessed quantitatively the efficiency of the LV localization task in Table 3. The studies in which available results can be compared with ours are highlighted in the table. All of them perform the LV localization task in fast-cine CMR sequences, while we do not find any attempts to localize LV from relaxometry acquisitions. Fast-cine sequences are designed to assess cardiac function by capturing the heart movement during the cardiac cycle. In these sequences, the LV shape changes over time while the other anatomical structures remain almost fixed. Hence, an OD algorithm designed to detect LV from fact-cine sequences resembles a motion detection algorithm able to identify a moving object on a fixed background. Other important CMR protocols, such as first-pass perfusion and relaxometry, are designed to characterize the myocardium tissue, with or without the use of a contrast medium. In these sequences, the acquisition is typically performed in the diastolic phase, avoiding heart motion. Hence, an OD algorithm designed to detect LV from these sequences should detect the signal change in LV over time due to the passing of a contrast medium (perfusion sequences) or the change of an MR acquisition parameter (TE for T2* and T2 relaxometry and TI for T1 relaxometry). LV detection from a relaxometry sequence represents a challenging task because the signal of all tissues in the field of view changes over time. Several papers in the literature deal with DL-based segmentation of CMR relaxometric sequences [34,35,36], but to the best of our knowledge, none of them included a preliminary LV localization task. The inclusion of an accurate cropping step based on LV localization may bring benefits even in segmentation due to the reduction in the computational space for subsequent operation.

We collected a global IoU of 0.68 and a CIR of 0.99. This means that the center of the bounding box predicted falls within the LV blood pool 99% of times. This could be particularly useful to automatically define a seed for any subsequent segmentation step in near real-time (20 ± 4 ms). The average IoU indexes in the three independent test sets were, respectively, 0.68 (T2*), 0.62 (T2), and 0.67 (T1), and the global CIR 0.94 (T2*), 0.95 (T2), and 0.98 (T1).

Several authors evaluated their performances according to the ability of the algorithm to predict the position of a centroid that falls within the LV blood pool, which we named CIR. By comparing our results reported in Table 1 with CIR obtained in relevant works we observed that our algorithm achieves state-of-the-art performances in localizing the LV centroid, both in global performances and in that ones differentiated by slice position [4,5,6]. We obtained a comparable DICE index, and a slightly worse $\epsilon_{CP,A}$, and $\epsilon_{CP,endo}$ with the ones reported by Tan et al. [6]. The authors localized the LV exploiting the heart’s motion. Cinetic information could not be exploited in our dataset as the T2* mapping protocol produces static sequences. The findings obtained highlight the great potential of deep learning models compared to the hand-crafted approaches.

As our dataset contains LV in all the input images, the developed model always returns as output a bounding box labeled as LV, and this would boost true and false positive and false negative rates and performances based on them such as Precision, Recall, and Average Precision (AP). The absence of slices without LV depends on the protocol employed to acquire the T2* sequences, which requires the acquisition of the three parallel short-axis views (basal, middle, and apical slices) of LV. We decided to not compute these metrics reporting only measurements of how close the bounding box predicted is to the GT bounding box, so our results are not comparable with the ones of Niu et al. [19], Wang et al. [21] and Shaaf et al. [8]. The only result comparable with the work of Shaaf et al. [8] is an averaged IoU over a cardiac cycle of 20 slices of 0.83, which authors reported as an Ovelap Ratio. We obtained a global IoU lower than the aforementioned work (0.68), but the authors reported only the results relative to a single acquisition, while our global IoU was obtained averaging 318 different images.

We used statistical analysis to verify if the performances evaluated differ according to slice type (basal, middle, or apical), as a smaller object (typically apical slices) could be potentially harder to be localized. Statistical tests confirm that ViT reported worse performances in performing localization in apical slices.

We selected the trained network which achieved the best results over the five different trials, and we applied it to an independent dataset of three different relaxometry sequences (T2*, T2, and T1). Statistical analysis did not highlight any significant difference in performances reported in the test set and in the T2* independent test set. This is reassuring since the two sets use the same mapping sequences. When applied to the T2 and T1, the independent test set statistical analysis highlighted lower performances as expected since the network had been training with different types of input. However, it should be noted that overall, the performances could be considered acceptable and CIR values remain consistent with relevant literature, with performances particularly promising for the T1 mapping.

Some limitations can be recognized in the present study. Firstly, training was performed on a dataset of limited size composed of a single scanner, a single bright-blood sequence dataset. Black-blood T2* images [37] were not included. Moreover, to match ViT’s architectures, we selected only 3 TEs from the entire multi-echo input sequence. All these aspects may affect the generalizability power of the developed model and the statistical significance of the results obtained. In the future, an extension of the study to increase the dataset’s dimension and also including multi-scanner acquisition could be advisable to overcome these limitations. A comparison of detection results obtained was performed with works that use a different type of CMR sequences (fast cine CMR), as we did not find any attempts to localize LV from relaxometry acquisitions in the literature. As our dataset contains LV in all the input images due to the standardized acquisition protocol, we could not assess the presence of LV differently from other works that performed both identification and location. Even if from a statistical point of view performances cannot be considered as perfectly generalizable to T2 and T1 mapping, considering its promising results, the present architecture could constitute a good starting point to apply transfer learning to T2 and T1 relaxometry sequences. So, future development of the work will include the fine-tuning of the present architecture on different types of relaxometry sequences.

## 5. Conclusions

In our research, we applied a ViT to automatically predict an ROI in CMR T2* mapping multi-echo sequences collected by the MIOT network. We evaluated detection performances performing k-fold cross-validation, and we differentiated them by slice view according to the AHA model. Once trained, we tested the model on an independent test set which included different types of mapping (T2*, T2, T1).

To the best of our knowledge, this is the first attempt to automatically detect LV from CMR relaxometry images using a ViT, a recently introduced deep learning architecture that may overcome different limitations of CNN models. All the other works in the literature performed the detection task on different types of imaging modalities, typically fast cine MR images. This study defines a benchmark in LV detection from CMR relaxometry sequences; with a CIR higher than those obtained in works that performed the same localization task but on different types of CMR acquisitions. The statistical analysis highlighted differences in the network’s performances according to the short axes view. In particular, we reported worse performances in apical slices; this may be due to the typically smaller size of the apical regions, which may result in being harder to be detected.

As results were collected on a dataset of relatively limited dimensions, in the future, an extension of the study will be performed to enhance the generalizability and statistical significance of the developed model. Performances were statistically different in the T2 and T1 independent sets but still promising considering that our network had been trained to detect LV from different types of imaging modalities.

The final trained model has been made publicly available and suitable for transfer learning. Future development will include the fine-tuning of the model on different relaxometric sequences.

## Figures and Tables

**Table 1:** Averaged object detection metrics (Mean ± St Dev) over the 5 folds evaluated in the test set.

Slice	IoU	DICE	$\epsilon_{\mathrm{CP},A}$	$\epsilon_{\mathrm{CP},\mathrm{epi}}$	$\epsilon_{\mathrm{CP},\mathrm{endo}}$	CIR
Basal	0.69 ± 0.03	0.81 ± 0.02	7.12 ± 0.97	0.27 ± 0.03	0.31 ± 0.04	1.00 ± 0.00
Middle	0.71 ± 0.02	0.82 ± 0.02	6.52 ± 0.81	0.26 ± 0.03	0.31 ± 0.04	0.99 ± 0.00
Apical	0.63 ± 0.01	0.77 ± 0.01	6.68 ± 0.77	0.33 ± 0.04	0.42 ± 0.05	0.97 ± 0.01
**Global**	0.68 ± 0.02	0.80 ± 0.02	6.77 ± 0.81	0.29 ± 0.03	0.35 ± 0.04	0.99 ± 0.00

**Table 2:** Averaged object detection metrics (Mean ± St Dev) over T2* test set, T2* independent test set, T2 independent test set and T1 independent test set.

Slice	IoU	DICE	$\epsilon_{\mathrm{CP},A}$	$\epsilon_{\mathrm{CP},\mathrm{epi}}$	$\epsilon_{\mathrm{CP},\mathrm{endo}}$	CIR
T2* test set						
Basal	0.74 ± 0.10	0.84 ± 0.07	5.44 ± 2.89	0.21 ± 0.11	0.24 ± 0.13	1.00
Middle	0.74 ± 0.10	0.85 ± 0.07	5.24 ±2.89	0.21 ± 0.12	0.25 ± 0.15	0.99
Apical	0.65 ± 0.13	0.78 ± 0.11	5.65 ± 3.45	0.29 ± 0.19	0.36 ± 0.24	0.97
**Global**	0.71 ± 0.12	0.82 ± 0.09	5.44 ± 3.10	0.23 ± 0.15	0.29 ± 0.19	0.99
T2* independent test set						
Basal	0.70 ± 0.15	0.81 ± 0.12	7.59 ± 6.33	0.25 ± 0.20	0.30 ± 0.23	1.00
Middle	0.69 ± 0.14	0.81 ± 0.12	7.42 ± 6.47	0.26 ± 0.21	0.30 ± 0.24	0.95
Apical	0.63 ± 0.21	0.76 ± 0.17	8.44 ± 7.08	0.36 ± 0.31	0.45 ± 0.38	0.86
**Global**	0.68 ± 0.17	0.79 ± 0.14	7.82 ± 6.54	0.29 ± 0.24	0.35 ± 0.30	0.94
**T2 independent test set**						
Basal	0.62 ± 0.12	0.75 ± 0.10	8.56 ± 5.67	0.29 ± 0.18	0.34 ± 0.21	1.00
Middle	0.62 ± 0.13	0.76 ± 0.10	9.32 ± 6.16	0.33 ± 0.21	0.39 ± 0.24	0.95
Apical	0.63 ± 0.14	0.76 ± 0.11	8.90 ± 6.02	0.38 ± 0.30	0.47 ± 0.38	0.90
**Global**	0.62 ± 0.13	0.76 ± 0.10	8.93 ± 5.86	0.33 ± 0.24	0.40 ± 0.29	0.95
**T1 independent test set**						
Basal	0.66 ± 0.09	0.79 ± 0.07	6.51 ± 2.90	0.22 ± 0.11	0.26 ± 0.12	1.00
Middle	0.69 ± 0.10	0.81 ± 0.07	6.38 ± 3.37	0.24 ± 0.16	0.28 ± 0.19	1.00
Apical	0.67 ± 0.13	0.80 ± 0.09	7.02 ± 4.57	0.31 ± 0.27	0.38 ± 0.34	0.95
**Global**	0.67 ± 0.11	0.80 ± 0.08	6.64 ± 3.63	0.26 ± 0.19	0.31 ± 0.24	0.98

**Table 3:** Performances of LV localization tasks in relevant works in literature. Performances comparable with ours are highlighted.

Author	Dataset	LV OD Approach	Performances
Pednekar et al. [4]	Vector Cardiographic Gating (VCG)-gated cine Steady-State Free-Precession SSFP cardiac MR images (locally acquired dataset)	Two different algorithms: Dual-contrast (DC) algorithm Scout-geometry (SG) algorithm	Best results obtained with SG algorithm. $CIR_{SG}=93\%$
Kurkure et al. [5]	Vector Cardiographic Gating VCG-gated cine SSFP cardiac MR images (locally acquired dataset)	Scout-view geometry, blood-to-myocardial tissue contrast, and geometrical continuity constraint	CIR=98.3%
Zhong et al. [17]	Cardiac cine MR images (locally acquired dataset), algorithm tested on 10 volunteers	Fourier Transform followed by anisotropic weighted circular Hough transform	By visual inspection, the cropped ROI of all cases contains the LV Average Cropping Ratio = 0.165
Tan et al. [6]	Cardiac cine MRI data: 161 clinical cine datasets (locally acquired) 1140 datasets from the Kaggle Datascience Bowl Cardiac Challenge 45 datasets from the STACOM 2009 Cardiac MR LV Segmentation Challenge	Fourier transform over time to highlight regions of significant motion	DICE: from 0.67 to 0.88 $\epsilon_{CP,A}:$ from 2.8 to 4.7 mm $\epsilon_{CP,endo}:$ from 12 to 22% CIR=97.3%
Niu et al. [19]	Cardiac cine MR images from Cardiac Atlas Project (CAP) dataset 27 subjects test set	Region proposal + Deep-Stacked Sparse Auto-Encoder (SSAE) + Soft margin support vector (C-SVC) classifier and multiple-output support vector (ϵ-SVR) regressor	F1=0.924 True positive rate, $T_{pr}=0.936$ Positive predicted value $P_{pv}=0.916$ AUC=0.89
Abdeltawab et al. [18]	Cardiac cine MR images: Locally acquired datasets ACDC-2017 MICCAI dataset	Fully Convolutional Neural network (FCN)	$\epsilon_{CP,A}$ = 1.41 ± 1.65
Wang et al. [21]	Cardiac cine MR images collected from CAP dataset	Two stage R-CNN based on VGG-16 backbone	Three different R-CNN version: Discriminant dictionary + Faster: AP50 = 91.86; AP75 = 84.17. Correlation filter + Faster: AP50 = 92.95; AP75 = 84.19. Correlation filter + Proposal: AP50 = 92.32; AP75 = 85.21.
Shaaf et al., 2023 [8]	Cardiac cine MR images collected from SCD MICCAI workshop 2009	Faster R-CNN	IoU = 0.83 Accuracy = 0.91 Recall = 0.95 Precision = 0.94 F1 = 0.95

## Data Availability

The data presented in this study are available on request from the corresponding author. The data are not publicly available due to privacy.

## Abbreviations

The following abbreviations are used in this manuscript:

SAV	Short Axis Views
LV	Left Ventricle
CMR	Cardiac Magnetic Resonance
AHA	American Heart Association
CV	Computer Vision
OD	Object Detection
ROI	Region Of Interest
DL	Deep Learning
AI	Artificial Intelligence
ACDC-2017	Automated Cardiac Diagnosis Challenge
FCN	Fully Convolutional Neural network
CAP	Cardiac Atlas Project
SSAE	deep-stacked sparse auto-encoder
R-CNN	Region-based Convolutional Neural Networks
YOLO	You Only Look Once
SSD	Single Shot Detector
SCD	Sunnybrook Cardiac Dataset
SSFP	Steady-State Free Precession
ViTs	Vision Transformers
NLP	Natural Language Processing
SA	Self Attention
DETR	DEtection TRansformer
COTR	COnvolutional and TRansformer layers
MIOT	Myocardial Iron Overload in Thalassemia
GT	Ground Truth
BT	Blood Pool
MHSA	Multi Head Self Attention
MLP	Multi-Layer Perceptron
IoU	Intersection over Union
CIR	Correct Identification Rate
